# Understanding the Salt-Dependent Outcome of Glycine Polymorphic Nucleation

**DOI:** 10.3390/pharmaceutics13020262

**Published:** 2021-02-15

**Authors:** Guangjun Han, Pui Shan Chow, Reginald B. H. Tan

**Affiliations:** 1 Institute of Chemical and Engineering Sciences, A-STAR (Agency for Science, Technology and Research), 1 Pesek Road, Jurong Island, Singapore 627833, Singapore; 2 Department of Chemical and Biomolecular Engineering, National University of Singapore, 10 Kent Ridge Crescent, Singapore 119260, Singapore; Reginald_Tan@hq.a-star.edu.sg

**Keywords:** solution crystallization, inorganic salt additives, polymorph control, glycine

## Abstract

The salt-dependent polymorphs of glycine crystals formed from bulk solutions have been a longstanding riddle. In this study, in order to shed fresh light, we studied the effects of seven common salts on primary nucleation of the metastable α-glycine and the stable γ-glycine. Our nucleation experiments and in-depth data analyses enabled us to reveal that (NH_4_)_2_SO_4_, NaCl and KNO_3_, in general, promote γ-glycine primary nucleation very significantly while simultaneously inhibiting α-glycine primary nucleation, thereby explaining why these three salts induce γ-glycine readily. In comparison, Ca(NO_3_)_2_ and MgSO_4_ also promote γ-glycine and inhibit α-glycine primary nucleation but not sufficiently to induce γ-glycine. More interestingly, Na_2_SO_4_ and K_2_SO_4_ promote not only γ-glycine but also α-glycine primary nucleation, which is unexpected and presents a rare case where a single additive promotes the nucleation of both polymorphs. As a result, the promoting effects of Na_2_SO_4_ and K_2_SO_4_ on γ-glycine do not enable γ-glycine nucleation to be more competitive than α-glycine nucleation, with γ-glycine failing to appear. These observations help us to better understand salt-governed glycine polymorphic selectivity.

## 1. Introduction

In the pharmaceutical industry, active pharmaceutical ingredients (APIs) are mostly crystals (crystalline materials) and they are usually produced via solution crystallization [1]. Given that crystals can have many polymorphs (i.e., crystalline structures or solid forms) and that the biological availability and other physicochemical properties [2] of polymorphs can largely differ, it is of paramount importance to control polymorphs in the manufacture of pharmaceuticals [2,3]. However, robust polymorph control has been a challenging task despite the great effort dedicated to fundamental explorations of crystal nucleation and growth [3,4,5,6,7,8,9], mainly because of the poor understanding of solution crystallization [3,8,9]. The challenges faced are perhaps particularly demonstrated by the longstanding puzzles [3,8,9,10,11,12,13,14,15] of glycine polymorphic crystallization from aqueous solutions with additives including inorganic bases, acids and salts.

Glycine (NH_2_CH_2_COOH), the simplest amino acid, is a classical polymorphic system which has played an important part in polymorphic studies. In an aqueous solution, glycine molecules exist as zwitterions (^+^NH_3_CH_2_COO^−^). In solid state under ambient conditions, it has three polymorphs [15]: the most stable γ-glycine (γ-form), the metastable α-glycine (α-form) and the least stable β-glycine (β-form), belonging to space group P3_1_ (or P3_2_), P2_1_/n and P2_1_, respectively (refer to Table S1 in the Supplementary Materials for more properties [16,17,18] of these three polymorphs). Glycine solids have applications in treating health problems [19]. Interestingly, it was found that, compared with α-glycine, γ-glycine exerts a beneficial effect on the behavior of catalepsy-prone rats [19], highlighting the importance of controlling glycine polymorphs.

An individual glycine polymorph from a bulk aqueous solution can be produced using various additives [8,9,12,20] or established techniques [21,22,23,24]. However, polymorphic crystallization of glycine is still studied extensively by many scientists [3,8,9,10,11,12,13,14,15,20,21,22,23,24,25,26]. One of the reasons [3,8,9] behind such extensive studies is that the mechanisms governing the outcome of glycine polymorphic crystallization may provide a clue for robust polymorph control of other polymorphic systems, given that glycine molecules in solution and in solid state exhibit many salient features.

**Figure 1:**
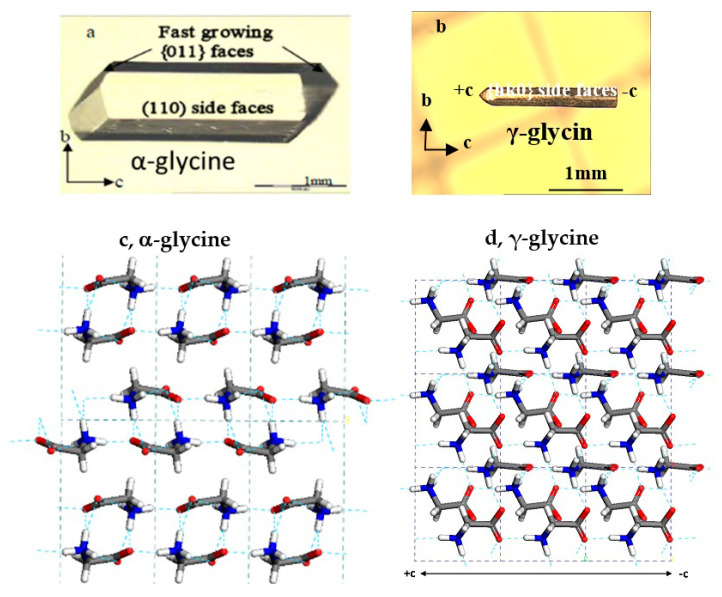
Typical habits (**a**,**b**) and different crystalline structures (**c**,**d**) (built using Materials Studio) of α-glycine and γ-glycine. Atom color scale in solid packing: C, dark gray; H, white; O, red; N, blue.

An early investigation [27] indicated that β-glycine, as the least stable form, practically does not have any chance to nucleate from an aqueous solution unless an unusually high supersaturation is established quickly [8,9] (which can be achieved using an antisolvent). Other previous studies [3,8,9] showed that the metastable dimer-based α-glycine (Figure 1) nucleates much faster than the stable monomer-based γ-glycine (Figure 1) from bulk pure (additive-free) aqueous glycine solution under usual conditions despite their thermodynamic stability, suggesting that α-glycine is kinetically favored. Furthermore, it was found that additives, including inorganic acids, bases and salts, can induce γ-glycine crystallization [8,9]. However, the mechanisms responsible for the additive-induced γ-glycine remain either unelucidated or debatable [8,9,12,15].

Acids and bases, acting as pH-regulating agents, cause glycine polymorphs to shift from the metastable α-glycine to the stable γ-glycine [3,8,9]. Over past decades, great attention has been paid to the long-standing riddle of pH-dependent polymorph switching [3,8,9]. Mechanistic exploration of this pH-governed polymorphic crystallization remains active [13]. Previous studies [3,8,9,13,28,29,30,31,32] provided insights into this riddle, contributing to the current understanding that glycine ions created at a high or low pH induce linear head-to-tail glycine chains that structurally favor the monomer-based γ-glycine.

Besides acids and bases, many common inorganic salts can also induce the stable γ-glycine [8,15]. However, compared with the extensive studies on pH effects on glycine polymorphs [9], far less attention has been paid to the effects of salts on polymorphic switching from α-form to γ-form. As it has been suggested [8,15], the salt-induced γ-glycine is hardly attributed to the pH shift, because many inorganic salts (e.g., KCl, KNO_3_ and NaCl) practically do not change the solution pH but they still induce γ-glycine. In addition, an early study [15] indicated that the growth rates of these two glycine polymorphs in the presence of various inorganic salts play a role but do not primarily determine the outcome of glycine polymorphic nucleation.

Studies of polymorphic transformation in bulk solutions [10,12] presented interesting results. It was shown that all the examined salts (KNO_3_, NaCl, (NH_4_)_2_SO_4_, Ca(NO_3_)_2_ and MgSO_4_) result in considerably shorter induction times of γ-glycine secondary nucleation during solution-mediated polymorphic transformation (SMPT) from α-glycine to γ-glycine. In other words, these salts promote the secondary nucleation of γ-glycine, with the monovalent cation salts ((NH_4_)_2_SO_4_, KNO_3_ and NaCl) being more effective than the divalent cation salts (MgSO_4_ and Ca(NO_3_)_2_). Another interesting observation [12,15] is that Ca(NO_3_)_2_ and MgSO_4_ promote γ-glycine secondary nucleation while they inhibit γ-glycine growth. Further analysis [12] suggests that inorganic salts may generally promote the primary nucleation of γ-glycine too, via ion-glycine interaction and formation of linear head-to-tail glycine chains which favor monomer-based γ-glycine.

A computational study [11] was also conducted to investigate how NaCl (1.37M) exerts different impacts on the nuclei of glycine polymorphs. It was suggested that Na^+^-based and Cl^−^-based double layers are established at the polar COO^−^-rich (00-1) and NH_3_^+^-rich (001) faces, respectively, at the c-axis of a γ-glycine nucleus. In comparison, similar double layers are also established around the faces of α-glycine but they are insignificant due to the low polarity of α-glycine faces. These appreciably different double layers alter the interfacial energies, increasing the nucleation barrier of α-glycine and reducing that of γ-glycine. As a result, NaCl retards α-glycine nucleation while accelerating γ-glycine nucleation, thereby inducing γ-glycine. However, an earlier study [8] had cast doubt on whether the formation of such double layers is a primary factor in directing the polymorphic crystallization, given that other salts (e.g., MgSO_4_ and Ca(NO_3_)_2_) can also form ion double layers but they hardly induce γ-glycine at a similar ionic strength.

In summary, the previous experimental and computational studies tend to suggest that inorganic salts, in general, favor γ-glycine over α-glycine to different extents. Two postulations were made to help elucidate the mechanisms: (1) inorganic salts promote γ-glycine primary nucleation and (2) they retard α-glycine primary nucleation. However, the validities of these two postulations have yet to be experimentally confirmed. Furthermore, it is a challenging task to experimentally investigate the effects of a salt on the coexisting courses of primary nucleation of both glycine polymorphs in the same solution, because usually only one polymorph appears and the other one does not appear via primary nucleation in a given single nucleation experiment. In fact, a general methodology is hardly available for one to assess the effects of a given additive on primary nucleation of both polymorphs. Moreover, extra caution has to be exercised when the salt-assisted primary nucleation of the stable γ-glycine is examined. This is because formation of pure stable γ-glycine can also originate from secondary nucleation in the presence of the earlier formed metastable α-glycine, which subsequently disappears through spontaneous α-to-γ transformation. All these challenges perhaps explain why experimental explorations of the effects of a given salt on primary nucleation of both glycine polymorphs are rare.

In this study, through properly designed solubility and nucleation experiments, we systematically investigated the effects of typical inorganic salts on primary (unseeded) nucleation of both γ-glycine and α-glycine. An in-depth analysis of the measured experimental data (e.g., nucleation temperatures and metastable zone widths) enabled us to extract more information on the effects of the examined salts on each individual glycine polymorph. To aid further analysis, experiments for SMPT from α-glycine to γ-glycine in the presence of each of the two particular salts, Na_2_SO_4_ and K_2_SO_4_, were also performed to examine their effects on γ-glycine secondary nucleation. The observed results are interesting and even surprising. Details of the results will be presented and discussed in the next sections.

## 2. Materials and Methods

### 2.1. Materials

The fine glycine crystals (99%), both α- and γ-glycine solid forms, were from Sigma-Aldrich (St Louis, MO, USA). Another glycine powder with higher purity (99.7%, Sigma-Aldrich) was used for control experiments. Seven analytical-grade salts were selected as the additives in glycine solution crystallization, with 5 of them (KNO_3_, NaCl, (NH_4_)_2_SO_4_, Na_2_SO_4_ and K_2_SO_4_) being monovalent cation ones and 2 (Ca(NO_3_)_2_*4H_2_O and MgSO_4_) being divalent cation ones. Ultrapure water (Millipore, Burlington, MA, USA, filtered with 0.22 μm pore size and deionized to approach a resistivity of 18.2 MΩcm) was used to prepare glycine–salt aqueous solutions.

### 2.2. Measurement of Solubility

In this study, glycine solubilities in solutions with inorganic salts at 25 °C and 28 °C were measured using a similar method [15]. The rationale for choosing this pair of temperatures will be discussed in Section 3.1 and Section 3.4.

The isothermal method for glycine solubility [15] is briefly described here. A suspension of a given solid form (either α- or γ-form) of glycine fine crystals was agitated in a jacketed beaker to establish the isothermal liquid-solid equilibrium at a temperature controlled by a Julabo FP50-HL circulator (Seelbach, Germanywith a temperature resolution of 0.01 °C). A precision densitometer (Anton Paar DMA5000, Graz, Austria) was used to regularly measure glycine solution density so as to determine solution concentration over a period of time (typically 30 min), until the solution was saturated, which was indicated by the unchanged glycine concentration (i.e., solubility) within a concentration uncertainty of 0.2%. The polymorphic form of the glycine crystals was examined before and after a solubility determination using powder X-ray diffraction (PXRD; refer to Figure S1 in the Supplementary Materials) (Bruker D8 Advance Diffractometer, Billerica, MA, USA).

### 2.3. Measurement of Nucleation Temperature and Metastable Zone Width

For each primary nucleation experiment, typically five runs were performed, with the average data of the measured nucleation temperature and metastable zone width (MZW) reported. In each run, a homogeneous aqueous glycine solution with a salt additive was prepared, with its glycine concentration corresponding to a saturation temperature of either 28 °C with respect to γ-glycine or 25 °C with respect to α-glycine.

A volume of 115 mL of the prepared solution was filtered through a 0.2-µm syringe filter into a jacketed beaker. This filtered sample solution in the beaker was kept stirred at 120 rpm using a magnetic stirrer. The jacketed beaker, parafilm-sealed to minimize evaporation, was connected to a water circulator (Julabo FP50-HL, Seelbach, Germany) to control the cooling rate of the sample solution. A Pt100 thermal probe was used to monitor the temperature of the sample solution in the jacketed beaker.

The solution temperature was increased to 33 °C (5 °C above the γ-glycine saturation temperature of 28 °C) and this temperature was maintained for 15 min for the homogeneous solution to attain thermal equilibrium. The sample solution was then cooled at a rate of 0.5 °C/min. The turbidity of the sample solution was monitored using a colorimeter (Brinkmann Instruments, New York, NY, USA). A sudden turbidity surge indicated the onset of glycine primary nucleation and, accordingly, the solution temperature was deemed to be the nucleation temperature. The metastable zone width (MWZ) was the difference between the saturation temperature (here, 25 °C if α-glycine nucleated and 28 °C if γ-glycine nucleated) and the nucleation temperature of a given glycine polymorph.

Once nucleation occurred, the temperature was maintained for a certain period of time to generate sufficient glycine crystals (>0.3 g) for subsequent polymorphic determination of the glycine solids via PXRD. On the other hand, this period of generating more crystals was also controlled to ensure that the concentration of glycine in the solution phase was higher than α-glycine solubility at the nucleation temperature. By doing so, any formed metastable α-glycine crystals were prevented from re-dissolution (disappearance) in the liquid phase even if the stable γ-glycine was also created, enabling us to capture the α-glycine crystals (if formed) for a proper analysis of the impacts of a salt additive on primary nucleation of individual glycine polymorphs.

Before the glycine crystals were harvested, a small sample (about 5 mL) of the suspension was taken to determine glycine concentration of the liquid phase using a solution densitometer (Anton Paar DMA5000, Graz, Austria) [15]. The rest (about 110 mL) of the suspension was then filtered quickly (in about 60 s) at ambient temperature (about 23 °C) under a vacuum using a ceramic Buchner funnel equipped with filter paper. The obtained solid glycine cake on the filter paper, typically about 1–2 g, was washed immediately using a solution saturated with pure α-glycine at ambient temperature to remove the salt from the glycine filter cake. This cake-washing step played a double role: (1) removing the salt on the crystal’s surface so as to effectively prevent α-glycine from being transformed to γ-glycine over the drying period, because such a transformation is very slow [12] in an additive-free environment; and (2) preventing glycine crystals in the glycine cake from being dissolved into solution during washing.

The wet glycine cake was then dried at ambient temperature. The mass of the dried glycine crystals was also measured to provide an alternative means of determining the glycine concentration of the liquid phase through mass balance. The polymorphs of the dried glycine solids were determined using PXRD.

This experimental procedure meant that metastable α-glycine crystals, if formed from solution, were supposed to be retained. In this case, if the obtained glycine solids were determined to be pure γ-glycine, then this indicated that the metastable α-glycine did not nucleate and hence the observed stable γ-glycine originated from γ-glycine primary nucleation.

### 2.4. Solution Mediated Polymorphic Transformation

An experimental procedure similar to that developed in our previous study [12] was used for SMPT where metastable α-glycine was transformed to stable γ-glycine in the presence of Na_2_SO_4_ and Ka_2_SO_4_ so as to obtain the induction times of γ-glycine secondary nucleation. For each SMPT experiment, typically 3 runs were performed, and the average induction time was obtained and used for further analysis.

The major steps of the experimental procedure are briefly described as follows. In each SMPT run, a solution was prepared, corresponding to α-glycine solubility at 23 °C. Next, 115 mL of this saturated glycine solution was transferred to a 250 mL jacketed beaker and its temperature was maintained at 23 °C through a water circulator. The sample solution was stirred at 120 rpm using a magnetic stirrer, then 10 g of fine α-glycine crystals was added to the sample solution. Slurry samples were taken regularly from the agitated solution using syringes. The glycine solid in a slurry sample, obtained through vacuum filtration, was rinsed and dried for PXRD analysis to detect the appearance of γ-glycine. An induction time of the secondary nucleation of the γ-form was defined as the time between the addition of α-glycine crystals and the appearance of γ-glycine.

## 3. Results and Discussion

### 3.1. Solubility Data

Glycine solubility in solutions with the selected salts at 25 °C and 28 °C was measured. The obtained solubility data of α-glycine and γ-glycine, typically having an uncertainty of ±0.2%, are presented in Table 1. They were used for preparation of glycine solutions in nucleation experiments.

To support a better analysis of glycine nucleation experiments, glycine solubilities at other temperatures were also measured and, accordingly, the relationships between glycine solubility and temperature at fixed concentrations of given inorganic salts were established. It was found that the solubilities of both α-glycine and γ-glycine increase linearly with temperature, having a common gradient of 0.48 ± 0.2 g/100 g H_2_O/°C in the temperature range of 10–28 °C. These measured solubilities, together with the established linear solubility-temperature relationships, enable us to reiterate that the metastable α-glycine has a higher solubility than the thermodynamically stable γ-glycine at a given temperature within the examined temperature range.

More interestingly, these inorganic salts cause the glycine solubility to increase, showing a salting-in effect. In particular, the salting-in effect of the divalent cation salts (Ca(NO_3_)_2_ and MgSO_4_) is significantly greater than that of the other salts examined here, partly due to a stronger interaction between divalent cation and glycine. Compared with the divalent cations (Ca^2+^ and Mg^2+^), the highly solvated divalent anion [15] SO_4_^2−^ (e.g., from Na_2_SO_4_, K_2_SO_4_ and (NH_4_)_2_SO_4_) exerted a less pronounced salting-in effect. The implication of the salting-in effect for glycine polymorphism will be further analyzed in Section 3.5.

The measured glycine solubilities at the 2 particularly chosen temperatures, 25 °C and 28 °C, have one salient feature. Perusal of the solubility data reveals that, at a fixed salt concentration of a given salt, α-glycine solubility at 25 °C and γ-glycine solubility at 28 °C are largely comparable. It is particularly true (Table 1) for the solubilities of these two glycine polymorphs in a pure solution and in solutions in the presence of KNO_3_, NaCl, (NH_4_)_2_SO_4_, Na_2_SO_4_ and K_2_SO_4_. This salient feature was exploited to extract extra nucleation information (refer to Section 3.4).

### 3.2. Effects of Salts on α-Glycine Primary Nucleation

Nucleation experiments were carried out to measure the nucleation temperatures and hence the metastable zone widths (MZWs) of glycine primary nucleation from solutions with various salts. The measured nucleation data enabled us to examine the effects of the salts on glycine primary nucleation, on the basis that a higher nucleation temperature or a narrower MZW means a faster nucleation rate.

As it was expected, the metastable α-glycine crystallized very readily, and γ-glycine did not appear during glycine primary nucleation from bulk pure (additive-free) aqueous solutions. Accordingly, the probability of α-glycine formation is 100% and that of γ-glycine formation is 0%. The typical nucleation temperature of α-glycine is 16.5 °C, which corresponds to an MZW of 8.5 °C (=25 °C–16.5 °C).

In the presence of the salts, the outcome of glycine polymorphic crystallization varied, depending on the salts and their concentrations. During cooling of a glycine solution, either α-glycine or γ-glycine appeared, and no mixture of α-glycine and γ-glycine was detected by PXRD. Furthermore, in the presence of a salt at its fixed concentration, usually only one highly dominant glycine polymorph was obtained, with the other polymorph being not competitive at all. However, there was an exception where, in the presence of 0.5 m NaCl, individual pure α-glycine (with a probability of 64%) from seven runs and pure γ-glycine (with a probability of 36%) from four runs was formed, out of 11 runs of glycine solution crystallization under practically the same conditions. The glycine polymorphs and the probabilities of their formation from primary nucleation in the presence of salts are summarized in Table 2.

Among the seven salts, four of them, namely Ca(NO_3_)_2_, MgSO_4_, Na_2_SO_4_ and K_2_SO_4_, did not induce γ-glycine even at a high salt concentration, with α-glycine being the only polymorph to appear. Note that the concentration of K_2_SO_4_ was only up to 0.5 m, limited by its solubility in water. It is therefore reiterated here that Ca(NO_3_)_2_ and MgSO_4_ do not induce γ-glycine, consistent with the previous observation [8]. However, in this current study, Na_2_SO_4_ did not induce γ-glycine either, different from the previous study [8], which revealed that γ-glycine together with α-glycine appeared during unseeded cooling solution crystallization in the presence of Na_2_SO_4_. This discrepancy will be discussed in Section 3.5.

As for KNO_3_ at a low concentration of 0.5 m, this did not lead to a glycine polymorphic shift from α-form to γ-form. In comparison, at a NaCl concentration of 0.5 m, either α-glycine or γ-glycine appeared. With increasing salt concentrations, KNO_3_ and NaCl induced γ-glycine readily, which will be elaborated in Section 3.3 and Section 3.4.

**Figure 2:**
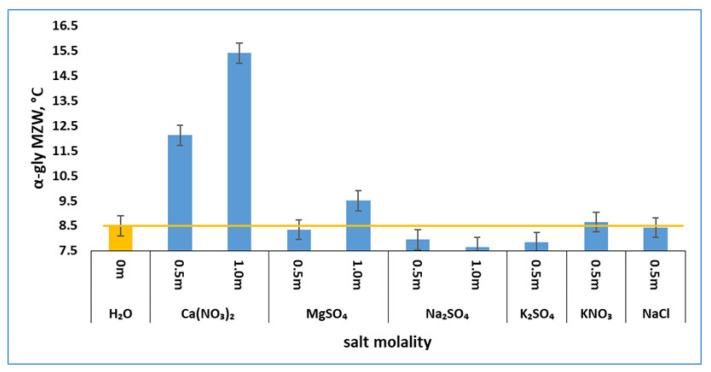
Impacts of four inorganic salts on the metastable zone widths (MZWs) of α-glycine primary nucleation (error bar = ±0.4 °C).

The measured MZWs of α-glycine primary nucleation in the presence of the salts are presented in Figure 2, showing the interesting effects of the salts. In a concentration range of 0.5–1.0 m, Ca(NO_3_)_2_ increased the MZWs of α-glycine primary nucleation, indicating that it inhibits α-glycine nucleation; MgSO_4_ at a low concentration of 0.5 m did not exert a significant effect on α-glycine while it inhibited α-glycine at a high concentration of 1.0 m. It is also observed that Ca(NO_3_)_2_ was far more effective than MgSO_4_ in inhibiting α-glycine primary nucleation. More interestingly, Na_2_SO_4_ and K_2_SO_4_ reduced MZWs (Figure 2), indicating that Na_2_SO_4_ and K_2_SO_4_ tend to promote rather than inhibit α-glycine primary nucleation, a surprising observation that is contrary to the previous postulation [10,11]. At a low concentration of 0.5 m, NaCl and KNO_3_ hardly changed the MZW of α-glycine, having insignificant and even negligible effects on α-glycine primary nucleation.

These MZWs (Figure 2) clearly reveal that these inorganic salts exerted mixed effects on α-glycine primary nucleation from a bulk solution. The inhibiting effect of Ca(NO_3_)_2_ and MgSO_4_ on α-glycine was expected, based on a previous report [10], which suggested that inorganic salts tend to weaken the formation of glycine cyclic dimers so as to hinder the primary nucleation of the dimer-based α-glycine. However, the observed promoting effect of Na_2_SO_4_ and K_2_SO_4_ on α-glycine is unexpected.

### 3.3. Effects of Salts on γ-Glycine Primary Nucleation

**Figure 3:**
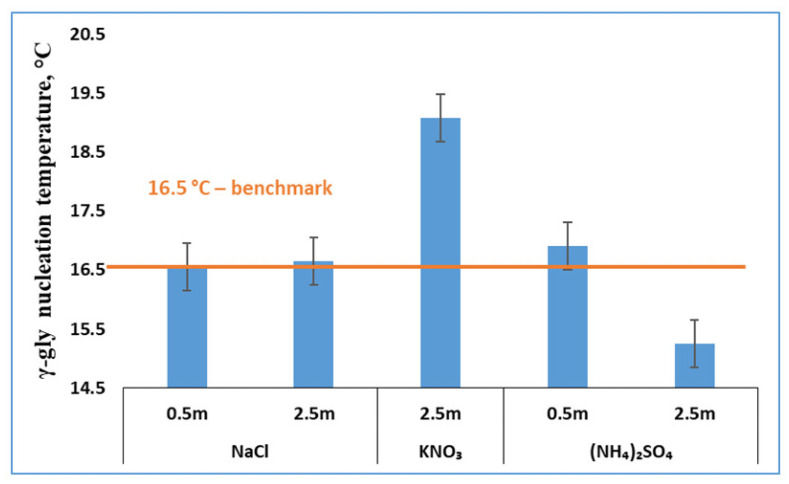
Effects of three salts on the nucleation temperature of γ-glycine (error bar = ±0.4 °C).

**Figure 4:**
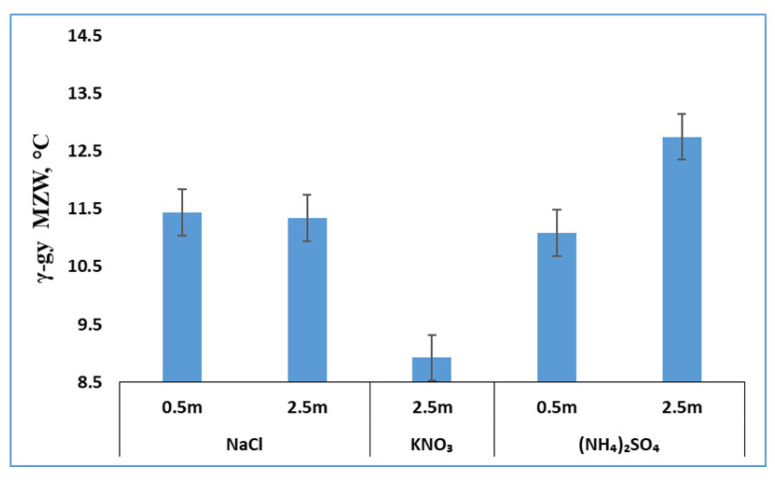
Effect of three salts on the MZWs of γ-glycine nucleation (error bar = ±0.4 °C).

In the presence of one of the three salts ((NH_4_)_2_SO_4_, KNO_3_ and NaCl), γ-glycine started to form during cooling, especially at a high salt concentration. In most cases, only pure γ-glycine appeared and α-glycine did not appear. As mentioned earlier, there was one exception that, in the presence of 0.5 m NaCl, either pure γ-glycine (with a probability of 36%) or pure α-glycine (with a probability of 64%) appeared at practically the same nucleation temperature of 16.5 °C. The nucleation temperatures of γ-glycine primary nucleation from various glycine solutions with these three salts were measured and the corresponding MZWs were calculated. To aid the subsequent discussion, both the nucleation temperatures and MZWs are presented in Figure 3 and Figure 4, respectively.

Over the period from nucleation onset to the point just before the harvest of the glycine crystals, the glycine concentration in the liquid phase was determined and it was found to be higher than α-glycine solubility at the nucleation temperature. Note that the α-glycine solubility at a nucleation temperature was predicted by our pre-established linear solubility–temperature relationships (Section 3.1). In this case, as analyzed earlier, if the metastable α-glycine was not detected and the stable γ-glycine was induced by the salts, then the salt-induced γ-glycine was supposed to originate from γ-glycine primary (rather than secondary) nucleation.

The formation of salt-induced γ-glycine is the outcome of relative competition between α-glycine and γ-glycine. In other words, neither salt-associated promotion of γ-glycine nor salt-associated inhibition of α-glycine can be concluded from the salt-assisted α-to-γ polymorph shift. In addition, since γ-glycine does not appear from a pure glycine solution, the quantitative MZW of γ-form from a pure solution is intrinsically unavailable. As a result, it is hard to determine whether a salt inhibits or promotes γ-glycine primary nucleation through a direct comparison between the MZW (Figure 4) of γ-glycine from a solution in the presence of a salt and that from a pure solution. It is seemingly even harder to assess if these three salts ((NH_4_)_2_SO_4_, KNO_3_ and NaCl) inhibit or promote α-glycine at the high salt concentrations at which α-glycine does not appear. Nevertheless, through our properly designed experiments and in-depth analyses of nucleation data of both α-form and γ-form, it is possible that the effects of a given salt on both α-glycine and γ-glycine can be inferred, with the details presented in Section 3.4.

### 3.4. Effects of Salts Inferred from In-Depth Data Analyses

As mentioned earlier, the metastable α-glycine crystallizes readily by cooling of a pure (additive-free) aqueous solution. This pure glycine solution has a saturation temperature of 25 °C with respect to α-glycine and a saturation temperature of 28 °C with respect to γ-glycine. Formation of the stable γ-glycine from the same pure aqueous solution was not observed, despite a number of experimental runs. Given the fact that, from a pure glycine solution, α-glycine is the highly dominant solid form (with a probability of 100%) and has a typical nucleation temperature of 16.5 °C, it is inferred that, even if γ-glycine eventually nucleates from the pure solution under the same experimental conditions, the nucleation temperature of γ-glycine has to be significantly lower than 16.5 °C. This characteristic temperature of 16.5 °C acts as a benchmark temperature that helps us to determine whether the three salts (NaCl, KNO_3_ and (NH_4_)_2_SO_4_) promote the primary nucleation of γ-glycine and inhibit that of α-glycine.

The glycine concentrations of the associated aqueous solutions in the presence of NaCl, KNO_3_ and (NH_4_)_2_SO_4_ practically also correspond to both a saturation temperature of 25 °C with respect to α-glycine and a saturation temperature of 28 °C with respect to γ-glycine, as reflected by the solubility data shown in Table 1. It follows that, during cooling, all these associated glycine–salt solutions would enter the first supersaturated region with respect to γ-glycine just below 28 °C and then the second supersaturated region with respect to α-glycine just below 25 °C, in the same manner as the corresponding pure glycine solution.

The particular feature that these associated glycine solutions have the same pair of dual saturation temperatures forms a fair basis, enabling us to compare the nucleation temperatures (Figure 3) of γ-glycine with the benchmark temperature of 16.5 °C to extract more nucleation information. If the nucleation temperature of γ-glycine from a salt solution is higher than the temperature benchmark of 16.5 °C, then the associated salt promotes γ-glycine primary nucleation, since γ-glycine can only nucleate from the corresponding pure glycine solution at a temperature much lower than 16.5 °C. According to the γ-glycine nucleation temperatures from various salt solutions (Figure 3), it is concluded that NaCl, KNO_3_ and (NH_4_)_2_SO_4_ in the indicated range of salt concentrations promote γ-glycine primary nucleation, with one exception, where the effect of 2.5 m (NH_4_)_2_SO_4_ on γ-glycine primary nucleation is undetermined.

Following a similar analysis, in the presence of a salt at a concentration higher than 0.5 m (except 0.5 m NaCl, at which both pure α-glycine and pure γ-glycine appeared from the respective batches), α-glycine never appears while γ-glycine is the dominant solid form. The practically zero probability of α-glycine formation means that even if α-glycine eventually nucleates from a salt solution under the same experimental conditions, α-glycine’s nucleation temperature should be significantly lower than the observed nucleation temperature of γ-glycine. It follows that, as long as the observed nucleation temperature (Figure 3) of γ-glycine from a salt solution is lower or even slightly higher (conservatively by 0.4 °C, the experimental uncertainty) than the nucleation temperature (16.5 °C) of α-glycine from the corresponding pure glycine solution, the nucleation of α-glycine from a salt solution is inhibited. It is therefore inferred that 2.5 m NaCl and 0.5–2.5 m (NH_4_)_2_SO_4_ (Figure 3) hinder α-glycine primary nucleation. The effect of 2.5 m KNO_3_ on α-glycine primary nucleation is undetermined.

Our inferences enabled us to confirm the salt-aided promotion of γ-glycine primary nucleation and/or inhibition of α-glycine primary nucleation in many more cases where the effects of salts on these two glycine polymorphs were otherwise not determined by the measured nucleation temperatures (and MZWs) themselves alone. The obtained inhibiting and promoting effects of various salts on α-glycine and γ-glycine primary nucleation, together with the results of salt-enhanced γ-glycine secondary nucleation during SMPT from an earlier study [12] and this study, are presented in Table 3, which aids further discussion and analysis.

In summary, our measured MZWs and in-depth data analysis reveal that these examined inorganic salts differed in their effects on α-glycine primary nucleation. While the majority of them exerted an inhibiting or insignificant effect on α-glycine, Na_2_SO_4_ and K_2_SO_4_ promoted α-glycine, a surprising observation. It is also confirmed that, in general, (NH_4_)_2_SO_4_, NaCl and KNO_3_ promoted γ-glycine primary nucleation. There are cases where the measured MZWs did not enable us to determine the effects (Table 3) of salts (especially Na_2_SO_4_, K_2_SO_4_, Ca(NO_3_)_2_ and MgSO_4_) on γ-glycine primary nucleation. These cases will be further analyzed in Section 3.5 with the data of the salt-enhanced γ-glycine secondary nucleation through SMPT, which, in fact, reveals that all the inorganic salts used here also generally accelerated the primary nucleation of γ-glycine.

### 3.5. Explanation of the Salt-Dependent Polymorphic Shift

As was observed from this study, the inorganic salts exerted mixed effects on the primary nucleation of α-glycine and γ-glycine. These results provide clues that help us to resolve the riddle that glycine’s polymorphic shift from α-from to γ-form is salt-dependent. In particular, both the inhibiting and promoting effects of the salts on α-glycine perhaps are more informative, and these different effects play roles in determining the outcome of glycine polymorphic nucleation from solutions with salt additives.

In a previous study [10], it was postulated that, in a glycine solution in the presence of a salt, the ion–dipole interaction between salt ions and glycine zwitterions is stronger than the dipole–dipole interaction between glycine zwitterions. This postulation is consistent with the observed salting-in effects (Table 1) on glycine solubility. The strong ion–dipole interaction tends to destroy the glycine cyclic dimers, which are deemed to be favorable building units of dimer-based α-glycine, suggesting that a salt hinders α-glycine formation. In this regard, the salt Ca(NO_3_)_2_ demonstrates the connection between the salting-in effect (arising from strong ion–dipole interaction) and inhibition of α-glycine formation. As observed, Ca(NO_3_)_2_ exerts a great salting-in effect (Table 1) on glycine solubility, significantly increasing the MZW (Figure 2) and thus suppressing the α-glycine primary nucleation rate at a high Ca(NO_3_)_2_ concentration of 1 m.

However, destroying glycine cyclic dimers by salt ions through strong ion–dipole interaction, thus disturbing α-glycine nucleation alone does not explain the observation that Na_2_SO_4_ and K_2_SO_4_ promote α-glycine primary nucleation. In particular, Na_2_SO_4_ promotes while (NH_4_)_2_SO_4_ inhibits α-glycine nucleation, despite their very similar salting-in effects (Table 1). The insignificant effect of Na_2_SO_4_ on α-glycine crystal growth [15] does not explain the Na_2_SO_4_-assisted promotion of α-glycine nucleation either. This suggests that there should be other factors favoring α-glycine nucleation. One such factor is the formation of different glycine–salt ion complexes in a solution of glycine and a salt additive, affecting the primary nucleation of each glycine polymorph.

In fact, previous studies [33,34] showed that glycine–salt crystals consisting of glycine and salt ions can be obtained through slow evaporation of a glycine–salt solution at a favorable glycine–salt molar ratio. It was reported [33] that in the presence of Ca(NO_3_)_2_, glycine–Ca(NO_3_)_2_-2H_2_O crystals were produced. The formation of such crystals may not be a surprise because divalent cation Ca^2+^ has a great capability for complexation with other molecules. It is, however, perhaps a surprise that even a monovalent ion salt (e.g., NaNO_3_) also leads to the formation of glycine–NaNO_3_ crystals [34]. Such observed glycine–salt crystals strongly indicate that glycine–salt complexes exist in solution. These glycine–salt complexes may act as nucleation transition states, either increasing or decreasing the α-glycine nucleation barrier depending on the charge, size and shape of the associated salt cations and anions, thereby exerting different impacts on α-glycine primary nucleation.

Similarly, through formation of various glycine–salt complexes in solution, the inorganic salts can promote or inhibit γ-glycine primary and secondary nucleation. In the current study (Table 3), salt-assisted promotion of γ-glycine primary nucleation was observed in several cases through measurements of MZWs, which is consistent with our previous study [12] where all the five examined salts ((NH_4_)_2_SO_4_, NaCl, KNO_3_, Ca(NO_3_)_2_ and MgSO_4_) promoted the secondary nucleation of γ-glycine (Table 3) during SMPT. To further support the idea that the inorganic salts as additives generally promote the secondary nucleation of γ-glycine so as to help reveal the effects of the salts (especially those that do not induce γ-glycine) on γ-glycine primary nucleation, in this study, glycine SMPT experiments were performed in the presence of Na_2_SO_4_ and K_2_SO_4_. It was found that Na_2_SO_4_ and K_2_SO_4_ also shortened γ-glycine induction times (from 15,640 min in the absence of salts to 55.2–59.1 min), promoting γ-glycine secondary nucleation (Table 3) by large enhancement factors of 265–283. (Note: an enhancement factor [12] is the ratio between induction times in the absence and in the presence of a salt.)

**Figure 5:**
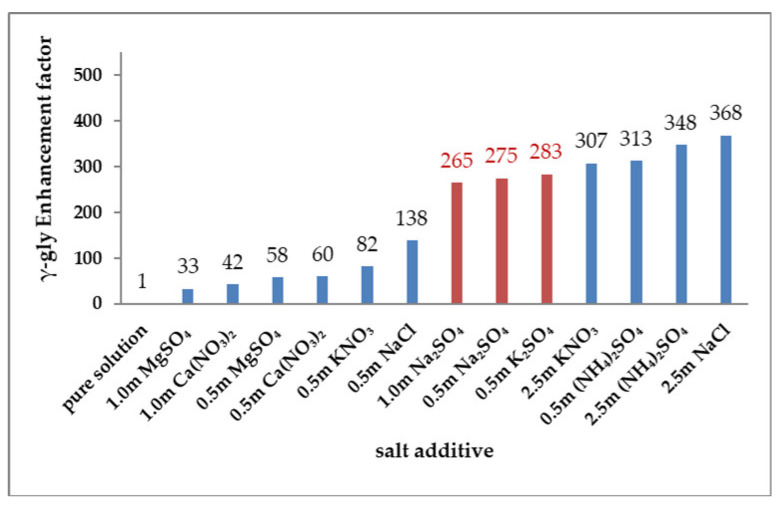
Salt-associated enhancement factors of γ-glycine secondary nucleation during solution-mediated polymorphic transformation (SMPT).

These new enhancement factors of γ-glycine secondary nucleation during SMPT in the presence of Na_2_SO_4_ and K_2_SO_4_, together with those reported in our earlier study [12], are presented in Figure 5. As shown in Figure 5, all the salts greatly promote the secondary nucleation of γ-glycine. Even the smallest enhancement factor at 1 m MgSO_4_ is up to 33 (which means 1 m MgSO_4_ increases γ-glycine secondary nucleation rate by 33 times). In fact, except Ca(NO_3_)_2_, MgSO_4_ and 0.5 m KNO_3_, all other salts exerted an even greater promoting effect on γ-glycine secondary nucleation, as evidenced by the corresponding enhancement factors, which are larger than 100 (Figure 5).

This generally observed salt-assisted enhancement of γ-glycine secondary nucleation (Figure 5) was explained based on a postulation [12] that the dissociated salt ions facilitate formation of the head-to-tail linear glycine chains among other glycine–ion complexes in a glycine solution. These formed linear glycine chains structurally favor γ-glycine, thereby likely playing a primary role in enhancing γ-glycine secondary nucleation. Based on this postulation, during unseeded primary nucleation, the dissociated salt ions can similarly induce the head-to-tail linear glycine chains in a glycine solution. Therefore, it is reasonable to suggest that these common inorganic salts, including Na_2_SO_4_ and K_2_SO_4_, generally promote γ-glycine primary nucleation too, which is also supported by the promoting effect of (NH_4_)_2_SO_4_, NaCl and KNO_3_ on γ-glycine primary nucleation in MZW experiments (Table 3).

Secondary nucleation and primary nucleation of γ-glycine differ. Compared with unseeded primary nucleation by cooling, γ-glycine secondary nucleation during a SMPT occurs in the vicinity of the α-glycine seed crystals (serving as a template favoring γ-glycine secondary nucleation) at a low supersaturation [12] (typically 1.06). Thus, at a fixed concentration of a given salt, the salt-associated enhancement of γ-glycine secondary and primary nucleation can be different to a certain extent. Nevertheless, when the enhancement factors (Figure 5) of γ-glycine secondary nucleation largely vary with salts, they are likely to reflect the salt-aided enhancements of γ-glycine unseeded primary nucleation to a significant extent, given the postulation that the linear glycine chains induced by the salt ions also play a primary role in enhancing γ-glycine primary nucleation.

Thus, perusal of the enhancement factors (Figure 5) and the results from primary nucleation (Figure 2 and Table 3) helps us to better understand the salt-dependent outcome of glycine polymorphic nucleation. Since, in pure (additive-free) glycine solutions, α-glycine strongly dominates γ-glycine, primary nucleation of α-glycine is supposed to be considerably easier than that of γ-glycine [12]. In order to induce γ-glycine, a salt additive has to favor γ-glycine primary nucleation over α-glycine to a great extent.

Generally, Ca(NO_3_)_2_ and MgSO_4_ inhibit α-glycine nucleation to a significant extent (Figure 2) while they strongly promote γ-glycine (Figure 5), but they only weaken the relative competition of α-glycine nucleation to a certain extent, and such a weakening of α-glycine nucleation is not sufficient to permit γ-glycine to appear. At a low concentration of 0.5 m, KNO_3_ promotes γ-glycine slightly more than Ca(NO_3_)_2_ and MgSO_4_ (Figure 5), but it affects α-glycine nucleation only insignificantly (Figure 2), generally failing to induce γ-glycine either.

In comparison, (NH_4_)_2_SO_4_ and NaCl generally accelerate the nucleation of γ-glycine to a greater (even far greater) degree (Figure 5). Given such promotion of γ-glycine nucleation, even if α-glycine nucleation is affected insignificantly at 0.5 m NaCl (Table 3), γ-glycine still starts to appear with a significant probability of 34%. Since 0.5–2.5 m (NH_4_)_2_SO_4_ and 2.5 m NaCl inhibit α-glycine nucleation, these salts induce γ-glycine even more effectively, causing γ-glycine to strongly dominate α-glycine. Similarly, concentrated KNO_3_ at 2.5 m also largely promotes γ-glycine, thereby inducing γ-glycine readily, though its effect on α-glycine is undetermined (Table 3).

The effects of these two particular salts, Na_2_SO_4_ and K_2_SO_4_, are different from all the other salts examined in this study. They promote primary nucleation of both α-glycine (Figure 2) and γ-glycine (as suggested from the aforementioned postulation that ion-induced linear glycine chains also enhance γ-glycine primary nucleation), presenting a rare and interesting case where a single additive enhances the primary nucleation of both polymorphs. The promoting effects of one sulfate salt on both glycine polymorphs cancel each other out to a certain extent. On a relative basis, this salt-aided acceleration of γ-glycine primary nucleation perhaps slightly weakens the competition of α-glycine primary nucleation. Nevertheless, α-glycine primary nucleation still remains more competitive than γ-glycine. As a result, Na_2_SO_4_ and K_2_SO_4_ fail to induce γ-glycine, even if they are similar to (NH_4_)_2_SO_4_ and NaCl, and enhance the primary nucleation of γ-glycine to a greater degree. This result is different from a previous observation [8] that a mixture of γ-glycine and α-glycine appeared during unseeded cooling solution crystallization in the presence of Na_2_SO_4_. Such a discrepancy may be attributed to the Na_2_SO_4_–enhanced secondary nucleation of γ-glycine (Figure 5), which occurred after the appearance of α-glycine crystals during the unseeded cooling solution crystallization in the previous study [8].

Compared with Na_2_SO_4_ and K_2_SO_4_, which promote both α-glycine and γ-glycine primary nucleation but fail to induce γ-glycine, the concentrated KNO_3_ at 2.5 m behaves both similarly and differently. On the one hand, it enhances γ-glycine nucleation to a comparable extent (Figure 5). On the other hand, it induces γ-glycine. Therefore, it is reasonable to suggest that KNO_3_ at 2.5 m is unlikely to promote α-glycine; otherwise, it fails to induce γ-glycine too.

It is necessary to reiterate that, in the presence of a given salt, different glycine–salt complexes exist in a glycine solution, exerting different impacts on α-glycine and γ-glycine primary nucleation. The net effect of a salt on primary nucleation of an individual glycine polymorph depends on the competition among the different impacts of the glycine–salt complexes that may be present in the solution. In order to confirm the existence and structures of glycine–salt complexes and quantify their contributions to the outcome of glycine polymorphic nucleation, an advanced computational study (e.g., molecular simulation) may be needed.

## 4. Conclusions

We performed experiments to investigate the effects of seven typical salts on the primary nucleation of α-glycine and γ-glycine. These salts exerted both promoting and inhibiting effects on α-glycine nucleation, while they all promoted γ-glycine nucleation, helping us to gain more insights into the salt-associated glycine polymorphism.

Here, MgSO_4_ and Ca(NO_3_)_2_ inhibited α-glycine while significantly promoting γ-glycine, but their effects were not sufficient enough to cause a polymorphic shift from α-form to γ-form. This is another reflection that, in pure additive-free glycine solutions, α-glycine nucleation strongly dominates γ-glycine nucleation.

Our comprehensive analyses of the nucleation data of both α-glycine and γ-glycine enable us to conclude that (NH_4_)_2_SO_4_, NaCl and KNO_3_ generally inhibit the primary nucleation of α-glycine while promoting that of γ-glycine to a greater extent than Ca(NO_3_)_2_ and MgSO_4_, thereby effectively inducing γ-glycine.

Furthermore, Na_2_SO_4_ and K_2_SO_4_ promote the primary nucleation of both α-glycine and γ-glycine, but α-glycine nucleation remains more competitive than γ-glycine. This promoting effect of these two salts on α-glycine is unexpected. This observation also presents a rare case where a single additive promotes both polymorphs.

The promoting effect of the salts on the primary nucleation of γ-glycine, revealed by MZW experiments (Table 3), is consistent with the postulation that [12] salt ions induce glycine head-to-tail chains structurally favoring γ-glycine formation. The inhibiting effect of the majority of these salts on α-glycine is also consistent with the hypothesis [10] that a salt tends to destroy cyclic dimers of glycine (acting as favorable α-glycine building units). However, the promoting effect of Na_2_SO_4_ and K_2_SO_4_ on α-glycine is against the above hypothesis, suggesting that more work needs to be done before the underlying mechanisms are elucidated at the molecular level.

Further study to examine the effects of inorganic salts on the nucleation of other polymorphic compounds is worthwhile, so that these simple common inorganic salts may be useful in general polymorph control in pharmaceutical and other industries.

## Figures and Tables

**Table 1:** Solubilities (g/100 g H_2_O) of glycine polymorphs in aqueous solutions with salt additives at 25 °C and 28 °C.

Salt Additive	Additive Concentration (Molality, m)	Ionic Strength of Solution	α-Glycine Solubility at 25 °C	γ-Glycine Solubility at 28 °C
NA	0.0	0.0	25.03	25.00
MgSO_4_	0.5	2.0	29.45	29.09
MgSO_4_	1.0	4.0	32.26	32.10
Ca(NO_3_)_2_	0.5	1.5	33.10	32.80
Ca(NO_3_)_2_	1.0	3.0	40.81	40.53
KNO_3_	0.5	0.5	26.84	26.80
KNO_3_	2.5	2.5	30.80	30.53
NaCl	0.5	0.5	25.98	25.98
NaCl	2.5	2.5	28.31	28.02
(NH_4_)_2_SO_4_	0.5	1.5	27.64	27.60
(NH_4_)_2_SO_4_	2.5	7.5	29.49	29.51
Na_2_SO_4_	0.5	1.5	27.47	27.45
Na_2_SO_4_	1.0	3.0	28.50	28.52
K_2_SO_4_	0.5	1.5	27.11	27.05

**Table 2:** The polymorphic outcome of glycine primary nucleation by cooling of its aqueous solutions with salt additives.

Salt Additive	Probability (%) of α-Glycine Formation	Probability (%) of γ-Glycine Formation
NA (additive-free)	100	0
0.5 m MgSO_4_	100	0
1 m MgSO_4_	100	0
0.5 m Ca(NO_3_)_2_	100	0
1 m Ca(NO_3_)_2_	100	0
0.5 m KNO_3_	100	0
2.5 m KNO_3_	0	100
0.5 m NaCl	64	36
2.5 m NaCl	0	100
0.5 m (NH_4_)_2_SO_4_	0	100
2.5 m (NH_4_)_2_SO_4_	0	100
0.5 m Na_2_SO_4_	100	0
1 m Na_2_SO_4_	100	0
0.5 m K_2_SO_4_	100	0

**Table 3:** Inhibiting and promoting effects of various salts on primary nucleation of α- and γ-glycine.

Salt	By MZW (Primary Nucleation)		By SMPT (Secondary Nucleation)
	α-Glycine	γ-Glycine	γ-Glycine
1 m MgSO_4_	inhibition	undetermined	promotion
1 m Ca(NO_3_)_2_	inhibition	undetermined	promotion
0.5 m MgSO_4_	insignificant	undetermined	promotion
0.5 m Ca(NO_3_)_2_	inhibition	undetermined	promotion
0.5 m KNO_3_	insignificant	undetermined	promotion
0.5 m NaCl	insignificant	promotion (inferred)	promotion
0.5–1 m Na_2_SO_4_	promotion	undetermined	promotion
0.5 m K_2_SO_4_	promotion	undetermined	promotion
2.5 m KNO_3_	undetermined	promotion (inferred)	promotion
0.5 m (NH_4_)_2_SO_4_	inhibition (inferred)	promotion (inferred)	promotion
2.5 m (NH_4_)_2_SO_4_	inhibition (inferred)	undetermined effect	promotion
2.5 m NaCl	inhibition (inferred)	promotion (inferred)	promotion

## Data Availability

All necessary data have been reported in this article and there are no other data to share.

## Supplementary Materials

The following are available online at https://www.mdpi.com/1999-4923/13/2/262/s1. Table S1: Properties of α-, β- and γ-glycine crystals. Figure S1: PXRD patterns of α-, β- and γ-glycine references (S1-a), α-glycine crystal samples (S1-b) before and after the α-glycine solubility test, and γ-glycine crystal samples (S1-c) before and after the γ-glycine solubility test.

## References

[1] Stoica C., Verwer P., Meekes H., Van Hoof P.J.C.M., Kaspersen F.M., Vlieg E. (2004). Understanding the Effect of a Solvent on the Crystal Habit. Cryst. Growth Des..

[2] Knapman K. (2000). Polymorphic Predictions. Mod. Drug Discov..

[3] Huang J., Stringfellow T.C., Yu L. (2008). Glycine Exists Mainly as Monomers, Not Dimers, in Supersaturated Aqueous Solutions: Implications for Understanding Its Crystallization and Polymorphism. J. Am. Chem. Soc..

[4] Black J.F.B., Cardew P.T., Cruz-Cabeza A.J., Davey R.J., Gilks S.E., Sullivan R.A. (2018). Crystal nucleation and growth in a polymorphic system: Ostwald’s rule, *p*-aminobenzoic acid and nucleation transition states. CrystEngComm.

[5] Yang H., Song C.L., Lim Y.X.S., Chen W., Heng J.Y.Y. (2017). Selective crystallisation of carbamazepine polymorphs on surfaces with differing properties. CrystEngComm.

[6] Chew J.W., Black S.N., Chow P.S., Tan R.B.H., Carpenter K.J. (2007). Stable polymorphs: Difficult to make and difficult to predict. CrystEngComm.

[7] Dowling R., Davey R.J., Curtis R., Han G., Poornachary S.K., Chow P.S., Tan R.B.H. (2010). Acceleration of crystal growth rates: An unexpected effect of tailor-made additives. Chem. Commun..

[8] Towler C.S., Davey R.J., Lancaster R.W., Price C.J. (2004). Impact of Molecular Speciation on Crystal Nucleation in Polymorphic Systems:  The Conundrum of γ Glycine and Molecular ‘Self Poisoning’. J. Am. Chem. Soc..

[9] Han G., Thirunahari S., Chow P.S., Tan R.B.H. (2013). Resolving the longstanding riddle of pH-dependent outcome of glycine polymorphic nucleation. CrystEngComm.

[10] Yang X., Lu J., Wang X.-J., Ching C.-B. (2008). Effect of sodium chloride on the nucleation and polymorphic transformation of glycine. J. Cryst. Growth.

[11] Duff N., Dahal Y.R., Schmit J.D., Peters B. (2014). Salting out the polar polymorph: Analysis by alchemical solvent transformation. J. Chem. Phys..

[12] Han G., Chow P.S., Tan R.B.H. (2016). Effects of Common Inorganic Salts on Glycine Polymorphic Transformation: An Insight into Salt-Dependent Polymorphic Selectivity. Cryst. Growth Des..

[13] Tang W., Mo H., Zhang M., Gong J., Wang J., Li T. (2017). Glycine’s pH-Dependent Polymorphism: A Perspective from Self-Association in Solution. Cryst. Growth Des..

[14] Ding L., Zong S., Dang L., Wang Z., Wei H. (2018). Effects of inorganic additives on polymorphs of glycine in microdroplets. CrystEngComm.

[15] Han G., Chow P.S., Tan R.B.H. (2016). Salt-dependent growth kinetics in glycine polymorphic crystallization. CrystEngComm.

[16] Iitaka Y. (1960). The crystal structure of β-glycine. Acta Cryst..

[17] Xavier N.F., Silva A.M., Bauerfeldt G.F. (2020). What Rules the Relative Stability of α-, β-, and γ-Glycine Polymorphs?. Cryst. Growth Des..

[18] Perlovich G.L., Hansen L.K., Bauer-Brandl A. (2001). The polymorphism of glycine: Thermochemical and structural aspects. J. Therm. Anal. Calorim..

[19] Markel A.L., Achkasov A.F., Alekhina T.A., Prokudina O.I., Ryazanova M.A., Ukolova T.N., Efimov V.M., Boldyreva E.V., Boldyrev V.V. (2011). Effects of the α- and γ-polymorphs of glycine on the behavior of catalepsy prone rats. Pharmacol. Biochem. Behav..

[20] Weissbuch I., Leisorowitz L., Lahav M. (1994). “Tailor-Made” and charge-transfer auxiliaries for the control of the crystal polymorphism of glycine. Adv. Mater..

[21] Aber J.E., Arnold S., Garetz B.A., Myerson A.S. (2005). Strong dc Electric Field Applied to Supersaturated Aqueous Glycine Solution Induces Nucleation of the γ Polymorph. Phys. Rev. Lett..

[22] He G., Bhamidi V., Wilson S.R., Tan R.B.H., Kenis P.J.A., Zukoski C.F. (2006). Direct Growth of γ-Glycine from Neutral Aqueous Solutions by Slow, Evaporation-Driven Crystallization. Cryst. Growth Des..

[23] Di Profio G., Tucci S., Curcio E., Drioli E. (2007). Selective Glycine Polymorph Crystallization by Using Microporous Membranes. Cryst. Growth Des..

[24] Kim J.-W., Shim H.-M., Lee J.-E., Koo K.-K. (2012). Interfacial Effect of Water/Oleic Acid Emulsion on Polymorphic Selection in the Cooling Crystallization of Glycine. Cryst. Growth Des..

[25] Vesga M.J., McKechnie D., Mulheran P.A., Johnston K., Sefcik J. (2019). Conundrum of γ glycine nucleation revisited: To stir or not to stir?. CrystEngComm.

[26] Meirzadeh E., Dishon S., Weissbuch I., Ehre D., Lahav M., Lubomirsky I. (2018). Solvent-Induced Crystal Polymorphism as Studied by Pyroelectric Measurements and Impedance Spectroscopy: Alcohols as Tailor-Made Inhibitors of α-Glycine. Angew. Chem. Int. Ed..

[27] Dang L., Yang H., Black S., Wei H. (2009). The Effect of Temperature and Solvent Composition on Transformation of β- to α-Glycine as Monitored in Situ by FBRM and PVM. Org. Process. Res. Dev..

[28] Han G., Poornachary S.K., Chow P.S., Tan R.B.H. (2010). Understanding Growth Morphology Changes of γ-Glycine and DL-Alanine Polar Crystals in Pure Aqueous Solutions. Cryst. Growth Des..

[29] Han G., Chow P.S., Tan R.B.H. (2012). Direct Comparison of α- and γ-Glycine Growth Rates in Acidic and Basic Solutions: New Insights into Glycine Polymorphism. Cryst. Growth Des..

[30] Hamad S., Catlow C.R.A. (2011). Are glycine cyclic dimers stable in aqueous solution?. CrystEngComm.

[31] Hamad S., Hughes C.E., Catlow C.R.A., Harris K.D.M. (2008). Clustering of Glycine Molecules in Aqueous Solution Studied by Molecular Dynamics Simulation. J. Phys. Chem. B.

[32] Chen J., Trout B.L. (2010). A Computational Study of the Mechanism of the Selective Crystallization of α- and β-Glycine from Water and Methanol−Water Mixture. J. Phys. Chem. B.

[33] Natarajan S. (1983). X-ray study and IR spectra of glycine calcium nitrate dihydrate. Z. Krist..

[34] Bhat M.N., Dharmaprakash S.M. (2002). New nonlinear optical material: Glycine sodium nitrate. J. Cryst. Growth.

